# Calorie-Restricted Mediterranean and Low-Fat Diets Affect Fatty Acid Status in Individuals with Nonalcoholic Fatty Liver Disease

**DOI:** 10.3390/nu13010015

**Published:** 2020-12-23

**Authors:** Danijela Ristic-Medic, Marijana Kovacic, Marija Takic, Aleksandra Arsic, Snjezana Petrovic, Marija Paunovic, Maja Jovicic, Vesna Vucic

**Affiliations:** 1 Group for Nutritional Biochemistry and Dietology, Centre of Research Excellence in Nutrition and Metabolism, Institute for Medical Research, University of Belgrade, 11000 Belgrade, Serbia; aleksandraarsicimi@gmail.com (A.A.); snjezana5.imr12@yahoo.com (S.P.); paunovic.marija90@gmail.com (M.P.); vesna.vucic.imr@gmail.com (V.V.); 2 Group for Immunology, Institute for Medical Research, University of Belgrade, 11000 Belgrade, Serbia; marijana.buac@imi.bg.ac.rs; 3 Group for Nutrition and Metabolism, Centre of Research Excellence in Nutrition and Metabolism, Institute for Medical Research, University of Belgrade, 11000 Belgrade, Serbia; marijapo2001@gmail.com; 4 Department of Gastroenterology, Clinical Hospital Center “Dr Dragisa Misović-Dedinje”, 11000 Belgrade, Serbia; maja.tufegdzic@gmail.com; 5 Euromedik Clinic, 11070 Belgrade, Serbia

**Keywords:** nonalcoholic fatty liver disease, steatosis, Mediterranean diet, low-fat diet, fatty liver index, HOMA-IR index, fatty acids

## Abstract

Lifestyle modifications are the main support of nonalcoholic fatty liver disease (NAFLD) therapy. Weight loss is one of the primary goals in NAFLD, but the effects of different calorie-restricted diets remain unclear. Thus, we evaluated the effects of two calorie-restricted diets—the Mediterranean diet (Med diet) and low-fat diet—on liver status, cardiometabolic markers, and fatty acid profiles in patients with NAFLD. Twenty-four overweight/moderately obese men were randomly assigned to consume one of these diets. Lipid levels, glucose, insulin, liver enzymes, steatosis, and fatty acid profiles of serum and erythrocytes phospholipids were assessed. After 3 months, all participants had a significant weight loss (>9%), with improvements in waist circumference, body fat %, index of visceral adiposity (VAI), lipid accumulation product, fatty liver (FLI), and hepatic steatosis (HSI) index (*p* < 0.001). Both diets significantly lowered triglycerides, total and LDL-cholesterol, liver enzymes, fasting glucose, insulin, and HOMA-IR index. Fatty acid profiles were enhanced after both diets, with a significantly decreased *n*-6/*n*-3 ratio. Participants on the Med diet had higher levels of HDL-cholesterol and monounsaturated and *n*-3 docosahexaenoic acids in serum phospholipids and lower levels of saturated fatty acids, triglycerides, TG/HDL ratio, and FLI when compared to participants on the low-fat diet. Our results indicate that dietary patterns and calorie restriction represent central therapeutic issues in the improvement of obesity-related cardiometabolic alterations that are involved in the mechanism of hepatic steatosis. The Med diet may contribute to disease treatment even more than the low-fat diet since it leads to decreased saturated and increased monounsaturated and *n*-3 polyunsaturated fatty acid status and improved FLI in NAFLD patients.

## 1. Introduction

The presence of obesity leads to a high-risk profile for the development of nonalcoholic fatty liver disease (NAFLD) [1]. Both an excessive body mass index (BMI) and visceral obesity are well-documented risk factors for NAFLD [2]. Imaging techniques such as liver ultrasonography are more sensitive tools for the diagnosis of liver steatosis in comparison to liver enzymes, which are elevated in NAFLD [2,3]. Besides the enzymes, high serum triglycerides and low HDL-cholesterol levels are also common in patients with NAFLD [4]. The prevalence of NAFLD in dyslipidemic individuals is about 50% [5]. According to the latest guidelines [2], the therapeutic approach to NAFLD is mostly focused on lifestyle interventions, including weight reduction, modification in dietary habits, and physical exercise [6]. The most recent not-double-blinded studies showed improvement in aminotransferases levels and liver steatosis by diets with different caloric restrictions and macronutrient compositions [2,7,8,9]. A weight loss of at least 5–7% has been shown to improve steatosis and also to reduce parameters of necrosis and inflammation in individuals with NAFLD, with greater improvements for higher weight loss [2,7].

The general recommendations for the diet are individualized, with a caloric deficit of 500−1000 kcal/day depending on the BMI of the NAFLD patient [2]. A reduced amount of total fats should constitute less than 30% of the total energy input, with a decrease in saturated fats and refined sugar intake and an increase in soluble fiber intake (low-fat diet). New evidence supports the idea that the Mediterranean (Med diet) may have an important role in the prevention and treatment of NAFLD [9,10,11].

Fatty acids (FAs) are important players in hepatic lipid metabolism [12]. In particular, *n*-3 polyunsaturated FAs (PUFAs), eicosapentaenoic acid (EPA, 20:5 *n*-3), and docosahexaenoic acid (DHA, 22:6 *n*-3) can increase fat oxidation and reduce endogenous lipid production at the gene level [13,14]. However, patients with NAFLD, especially obese ones, mostly have an impaired *n*-3 PUFA status and a high *n*-6/*n*-3 ratio that can be improved by a proper diet [15,16]. Furthermore, pooled estimates of case–control studies have shown that blood and/or liver DHA contents are lower in NAFLD patients [17].

Only limited information exists on the interaction between different diets and fatty acid profiles in NAFLD patients. It has been established that the male gender is a risk factor for NAFLD [18]. Therefore, the aim of this study is to evaluate the effects of two personalized calorie-restricted diets—the Med diet and the low-fat diet—on liver status (evaluated by liver ultrasonography and biochemical parameters) and phospholipid fatty acid profiles of serum and erythrocytes in male patients with NAFLD after a 3-month follow-up.

## 2. Materials and Methods

### 2.1. Study Participants

**Figure 1:**
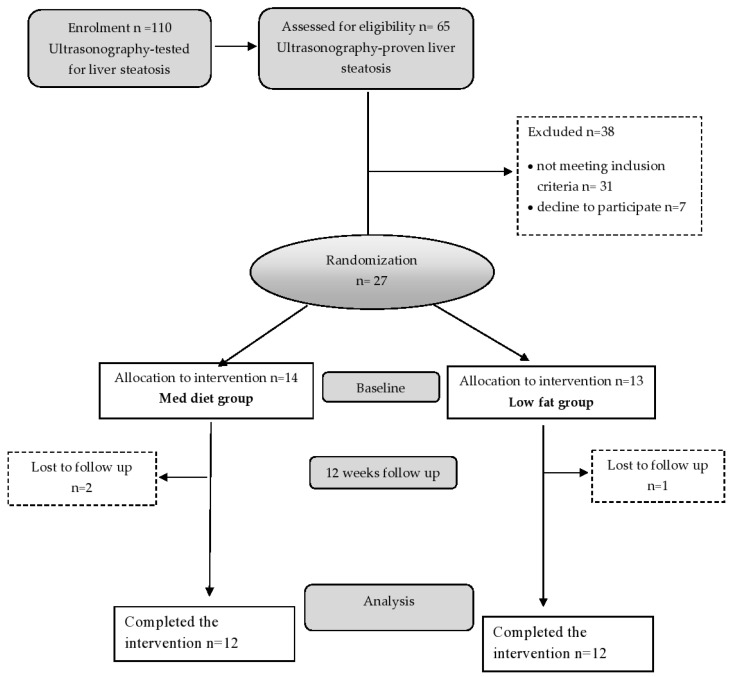
Flow chart of the study participants.

Twenty-seven overweight or moderately obese men with a body mass index (BMI) of 25 to 35 kg/m^2^, 27–42 years of age, with ultrasonography-diagnosed NAFLD, were enrolled in this study (Figure 1). All participants received a full medical examination and performed standard blood tests before enrolment. Participants were recruited at the Centre of Research Excellence in Nutrition and Metabolism, Institute for Medical Research, from February 2013 to March 2015. Randomization was performed using block-balanced randomization in a 1:1 ratio into the Med diet and low-fat diet groups using a computer-generated sequence. The inclusion criteria were adult male patients, overweight or moderately obese, with ultrasonography-confirmed NAFLD. Subjects who had evidence of any liver disease other than NAFLD, diabetes mellitus, thyroid disease, serious gastrointestinal disease, cardiac, renal, or active autoimmune disease were excluded from the study. The exclusion criteria also involved alcohol consumption (>20 g/day), smoking habits, and using hepatotoxic drugs. Weight loss of 3 kg or more in the last 3 months, taking antioxidant supplements or fish oil/omega (3 supplements), or high intakes of fish, flaxseed, or walnuts (more than 2 servings per week) were also exclusion criteria. The study was approved by the Ethics Committee of the University of Belgrade (Ethics number 36/18/2011), in accordance with the principles of the Declaration of Helsinki, as part of a research project of the Ministry of Science of the Republic of Serbia (III41030).

### 2.2. Study Design

The study design and flow chart of the study are presented in Figure 1. Participants were randomly assigned to consume a calorie-restricted (daily 600–800 kcal intake reduction) diet in the form of either the standard Med diet or a low-fat diet for 3 months. All participants received personalized nutritional counseling, and a daily dietary treatment, with 30% caloric restriction from the estimated daily energy requirements, was prescribed individually to achieve a loss of at least 5% of initial body weight. The daily energy requirements were calculated according to obesity treatment guidelines issued by the Board of the US National Institute of Health and Food and Nutrition [19]. The conventionally balanced distribution of macronutrients in the Med diet is 50% of the total caloric value from carbohydrates, 15% from proteins, and above 30% from lipids. In the low-fat diet, established macronutrient distribution was 60% of the total caloric value from carbohydrates (low glycemic index), 15% from proteins, and less than 25% from lipids. The calorie-restricted diets were also balanced in terms of micronutrient intake (vitamins and minerals in the recommended diet must always be checked). Both diets were designed to include breakfast, lunch, dinner, one snack in the morning, and one snack in the afternoon. Dietary intake was controlled by the size of portion, food choice, and composition. Participants in the calorie-restricted diet groups were educated by our medical dietitian on the principles of how to reduce daily energy intake and achieve weight reduction. Information concerning habitual dietary intake was obtained at the beginning of the study using a validated semiquantified food frequency questionnaire (FFQ). All participants in both intervention groups received personalized nutritional counseling by a professional nutritionist. The two diets differed in nutritional composition, e.g., macronutrients, fiber, and antioxidants, as presented in Table S1. Briefly, the Med diet is rich in vegetables, with increased poultry and fish intake and limited red meat consumption. Animal fats such as butter, cream, and lard are not allowed in this diet [11]. Participants assigned to the low-fat diet were advised to reduce their intake of all types of fats from both animal and vegetable sources. All participants in both groups were also advised to increase their level of physical activity by walking 30 min per day.

### 2.3. Biochemical Analysis

Blood samples for all biochemical analyses were obtained at the beginning and the end of the treatment, in the morning, after an overnight fast. Fasting glucose, serum lipids triglycerides (TGs), total cholesterol, HDL-cholesterol, LDL-cholesterol) and liver enzymes (aspartate aminotransferase (AST), alanine aminotransferase (ALT), and gamma-glytamiltransferase (GGT)) levels were determined on the same day the samples were collected using a clinical chemistry automated analyzer (Cobas c111, Roche Diagnostics, Basel, Switzerland) and commercial Roche diagnostics kits, according to the manufacturer’s instruction. Insulin level was measured using the radioimmunoassay method (INEP Zemun, Belgrade, Serbia). Fasting insulin and glucose concentrations were used to calculate insulin resistance from the HOMA-IR model (insulin × glucose/22.5). Hs-CRP was measured by the Olympus (LATEX) assay on the Olympus AU 400 analyzer (Olympus, Center Valley, PA, USA).

### 2.4. Anthropometric Parameters and Calculated Indexes

The anthropometric measurements included height, weight, waist circumference, and body fat. Waist circumference was measured from the midpoint between the lateral iliac crest and the lowest rib to the nearest 0.5 cm. Height was measured to the nearest 0.5 cm using a wall-mounted stadiometer. Body weight and body fat percentage were measured using a Tanita body composition analyzer (model TBF–300, Tokyo, Japan) to the nearest 0.1 kg, with the subject wearing light clothes, without shoes. Body mass index (BMI) was calculated as weight (kg)/height (m) squared. Abdominal ultrasonography, as a first-line investigation for hepatic steatosis, was performed on all patients by the same operator, who was unaware of the clinical and laboratory results, at the start and the end of the study. NAFLD was diagnosed by standardized criteria [20].

The visceral adiposity index (VAI) was calculated using the formula [21]:

VAI = [(waist circumference (cm)/39.68 + (1.88 × BMI)) × (TG (mmol/L)/1.03) × (1.31/HDL (mmol/L)](1)

The fatty liver index (FLI) was calculated according to the formula [22]:

FLI = (e ^0.953*loge (TG (mg/dL) + 0.139*BMI (kg/m^2^) + 0.718*loge (GGT) + 0.053* waist circumference (cm)−15.745^)/(1 + e ^0.953*loge (TG mg/dL) + 0.139*BMI (kg/m^2^) + 0.718*loge (GGT) + 0.053*waist circumference (cm)−15.745^) * 100 (2)

Fatty liver index <30 excludes liver steatosis, whereas FLI ≥ 60 confirms hepatic steatosis detected by ultrasonography [23].

Two other indexes, the hepatic steatosis index (HSI) [(8 × 9 ALT/AST) + BMI] and the lipid accumulation product index [(waist circumference (cm) − 65) × TG (in mmol/L)] were also calculated for the assessment of liver steatosis [24,25].

### 2.5. Fatty Acid Analysis

As previously described, serum lipids and erythrocyte membrane lipids were extracted with a chloroform–methanol mixture (2:1 *v*/*v*) according to the methods of Sperry et al. and Harth et al., respectively [26]. The phospholipid fraction was isolated by one-dimensional thin-layer chromatography, and direct transesterification of phospholipid FAs was applied. Fatty acid methyl esters were analyzed by gas–liquid chromatography on the Shimadzu chromatograph GC 2014 (Kyoto, Japan), equipped with a flame ionization detector on an Rt × 2330 column (60 m × 0.25 mm ID, film thickness of 0.2 μm; RESTEK, Bellefonte, PA, USA). The identification of fatty acid methyl esters was made by comparing peak retention times with standard mixtures (PUFA−2 and/or 37 FAMEs mix; Supelco, Bellefonte, PA, USA); FA is presented as a percentage of total FAs identified.

### 2.6. Sample Size Calculation

The sample size was calculated with triglycerides as primary outcomes based on a study by Abenavoli et al. [27] to detect differences of 55 mg/dL for TGs between study groups, with a 95% confidence interval (α = 0.05) and a statistical power of 80% (β = 0.8). The calculation estimated 11 participants per study group but expected a dropout rate of 10–20%; we included 13–14 patients per group.

### 2.7. Statistical Analysis

The normality of the distribution of variables was checked using the Shapiro–Wilk test. Normally distributed data are shown as mean values ± standard deviation (SD), while data with non-normal distribution (triglycerides, AST, HOMA-IR, CRP, docosatetraenoic acid, ALA, EPA, and MUFA/PUFA ratio in serum and *n*-6/*n*-3 and MUFA/*n*-3 PUFA in erythrocytes) are presented as median values and interquartile ranges. The differences between baseline and after the treatment period (intragroup comparisons) were determined by paired Student’s *t*-test (normally distributed) and Wilcoxon test (nonparametric data), while differences between the 3-month changes induced by the two diets were calculated by ANCOVA adjusted for baseline values. Univariate linear regression analyses were used to evaluate the potential association between anthropometry, clinical data, and biochemical parameters of the diet and the hepatic status variables. To provide an adjusted intergroup analysis, multivariable linear regression analyses were applied and adjusted for age and biochemical parameters, which showed significant differences in previous analyses (AST, HDL-cholesterol, and triglycerides). Analyses were carried out using the SPSS 20 program (IBM, Armonk, NY, USA). A *p*-value < 0.05 indicates statistical significance.

## 3. Results

The anthropometric and clinical characteristics of the study participants are presented in Table 1. Study participants had BMIs of about 30 kg/m^2^ and waist circumferences >94 cm. At the start, there were no differences in mean age, weight, BMI, waist circumference, body fat percentage, VAI, FLI, and HSI between the groups. All the parameters were significantly improved after 12 weeks on the Med or low-fat diet (*p* < 0.001 compared to baseline). In terms of the FLI index, both diets were very effective, decreasing the index markedly in all study participants. Comparison of the efficacy of the two diets showed that the Med diet reduced FLI more than the low-fat diet. In addition, at the end of the study, six men from the Med group and eight men from the low-fat group had ultrasonographic findings of NAFLD.

Table 2 shows biochemical parameters before and after Med or low-fat diet in NAFLD patients. There was n difference in the baseline levels of serum biochemical parameters, HOMA index, TG/HDL-cholesterol ratio, and lipid accumulation product index. After 12 weeks, both diets significantly reduced triglycerides, total and LDL-cholesterol, fasting glucose, fasting insulin, and hs-CRP concentration and activities of liver enzymes (ALT, AST, GGT). Consequently, lipid accumulation product index, HOMA-IR index, and TG/HDL-cholesterol ratio decreased (*p* < 0.001 compared to baseline) after treatments. In Med and low-fat diet groups, TGs severely decreased by 1 and 0.83 mmol/L and the TG/HDL-cholesterol ratio by 0.85 and 0.75, respectively. In the Med diet group, HDL-cholesterol levels increased more than in the low-fat diet group (*p* < 0.05). Additionally, in the intergroup comparison, in the Med diet patients, we found more exerted reductions of triglycerides, TG/HDL-cholesterol ratio, AST, and lipid accumulation product index, as well as increased HDL-cholesterol, than in the low-fat diet group.

At the baseline, there were no significant differences in the percentage of FAs in serum phospholipids between the two study groups (Table 3). The Med diet induced a significant decrease in saturated FA 16:0 and total saturated fatty acid (SFA) levels compared to baseline and the low-fat group. Levels of EPA, DHA, and total *n*-3 PUFA significantly increased after both diet treatments. Consequently, the *n*-6/*n*-3 total PUFA ratio decreased in both groups. Unlike the Med group, the low-fat group demonstrated decreased oleic acid (18:1 *n*-9, *p* < 0.05) and total MUFAs (*p* < 0.05) in serum phospholipids. Accordingly, after the low-fat diet, the MUFA/*n*-3 and MUFA/PUFA ratios decreased compared to baseline, while in the Med group, MUFA/SFA and MUFA/*n*-6 increased and MUFA/*n*-3 decreased significantly. In addition, the change from baseline to the endpoint was significantly different in proportions of palmitic acid and SFA, which were higher in the low-fat diet group than in the Med diet group, and oleic acid, MUFA, DHA, and MUFA/SFA ratio, which were all higher in the Med diet group at the end of the treatment than in the Low-fat group (Table 3).

The effects of the diets on the percentage of FAs in erythrocyte phospholipids are given in Table 4. Both diets significantly increased DHA and total *n*-3 PUFAs, while the *n*-6/*n*-3 PUFA ratio decreased. The Med diet also increased the percentage of oleic acid, total MUFAs (*p* < 0.01), as well as the MUFA/SFA (*p* < 0.01) and MUFA/*n*-6 ratios (*p* < 0.05). At the end of the treatment, 16:0 and total SFAs were higher and *n*-6 PUFAs and total PUFAs were lower in the low-fat diet group compared to the Med diet group. Nevertheless, no significant differences were found when we compared 3-month differences in the Med group with 3-month differences in the low-fat group using ANCOVA.

Linear regression analyses were performed to assess the factors that could influence the markers of hepatic status after 3 months of the Med and low-fat diets (Table S2). In the Med diet, the decrease in body fat percentage was significantly associated with improvements in hepatic steatosis markers: VAI, FLI, lipid accumulation products, AST, and triglyceride level. The same predictor was significantly associated with a decrease in AST and ALT in the low-fat group. Reduced waist circumference was associated with improved HSI and ALT in the low-fat diet group. Further, changes in HOMA-IR were associated with a reduction of VAI, FLI, lipid accumulation products, and triglycerides in the Med diet group and with VAI in the low-fat diet group. The changes in FA status showed no significant association with the parameters of NAFLD (data not shown). Multiple linear regression analysis was carried out to assess the influence of different types of diets on the decrease in FLI, adjusted for age, and biochemical parameters AST, HDL, and TGs, which showed significant differences from the previous analyses (Table 5). The results have shown that the decrease of FLI was statistically and significantly associated with the type of diet, demonstrating that persons on the Med diet will have a greater reduction of FLI than those who are adherent to the low-fat diet (β = 0.377; *p* = 0.043; adjusted R^2^ = 0.103). Model 2 has shown that the type of diet influences a decrease in FLI independently of age (β = 0.452; *p* = 0.023), while Model 3 has demonstrated that biochemical parameters AST, HDL, and triglycerides did not affect FLI changes (β = 0.466; *p* = 0.032).

## 4. Discussion

Nutrition and dietary patterns have been proposed as potential environmental factors that can affect the development of NAFLD. The basic recommendation for individuals with NAFLD is lifestyle modification, including dietary interventions. However, the evidence for nutritional factors, dietary characteristics, and dietary strategies for NAFLD treatment remains inconclusive. In the present study, we have shown that at a 3-month follow-up, both calorie-restricted dietary treatments, the Med diet and a low-fat diet, in male overweight or moderately obese participants with NAFLD induced (1) overall decreases in total cholesterol, triglycerides, TG/HDL-cholesterol ratio, glucose, hs-CRP, and liver enzymes levels; (2) significant weight loss (>9%); (3) improvement in ultrasonography findings, body fat percentage, waist circumference, VAI, FLI, HSI, and lipid accumulation index; (4) increased *n*-3 PUFAs levels in serum and erythrocyte phospholipids. Our results confirm that dietary patterns represent a central therapeutic issue in the improvement of obesity-related metabolic alterations involved in the mechanism of hepatic steatosis. Moreover, the Med diet leads to greater improvements of ALT, HDL-cholesterol, triglycerides, TG/HDL-cholesterol ratio, lipid accumulation products, FLI, and DHA and MUFAs content in serum phospholipids compared to a low-fat diet.

Both calorie-restricted dietary treatments in our study resulted in more than 9% of body weight loss. Weight loss remains the cornerstone of therapy in NAFLD patients. Findings from meta-analyses suggest that ≥5% of weight loss may have beneficial effects on liver and cardiometabolic parameters and steatosis. Additionally, ≥7% of weight loss improves histological disease activity in NAFLD and liver injury (plasma ALT, AST), which is in accordance with our results [7,28]. In regression analyses, a decrease of body fat percentage at the endpoint of the intervention was associated with improved VAI, FLI, lipid accumulation products, and ALT in the participants on the Med diet and also with ALT and AST improvements in those on the low-fat diet. Furthermore, reduced waist circumference in the low-fat diet group has shown associations with HSI and ALT. The improvement in steatosis index in both diet groups is an important indicator of lifestyle modification benefits in our NAFLD patients. FLI, HSI, and lipid accumulation product index, as steatosis biomarkers, are helpful in the clinical assessment of the severity of liver steatosis [22,23,24,25]. The group on the Med diet had a more pronounced improvement in FLI; 50% of the patients (6 out of 12) lost the ultrasonography findings of NAFLD, which is in accordance with a previous report [6,27]. Multivariate regression analysis has shown that a decrease in FLI is greater after the Med diet, independent of age and biochemical parameters of the study participants.

Although both diets improved lipid status in our NAFLD patients, the Med diet led to a higher increase of HDL-cholesterol and a decrease in triglycerides and the TG/HDL cholesterol ratio. Lower levels of circulating triglycerides, which we detected after the dietary treatments, may be due to a lower synthesis of hepatic triglycerides, and/or lower secretions of very low-density lipoprotein (VLDL)-TGs in plasma. It is considered that an imbalance between the intrahepatic production of triglycerides, and the removal of intrahepatic triglycerides is the basis for the accumulation of fat in the liver [29]. The increase in VLDL-TG secretion, which is the major source of circulating triglycerides, is most probably responsible for the higher levels of triglycerides, commonly observed in patients with NAFLD [30]. Furthermore, patients with NAFLD have an increased expression of HMG-CoA reductase and, consequently, increased synthesis of hepatic cholesterol [31]. Several studies have confirmed that NAFLD is associated with elevated fasting cholesterol, TG and LDL concentrations, and lower HDL-cholesterol concentration, which is in accordance with our results [31,32,33]. This atherogenic lipid profile and higher TG/HDL-cholesterol ratio can be associated with different pathologies such as insulin resistance, obesity and metabolic syndrome, commonly found in NAFLD patients [34].

Besides lipids, the serum concentration of liver enzymes markedly decreased after the diets in patients with NAFLD, and AST was even more reduced after the Med diet than at the end of the low-fat diet. These findings are, in agreement with other authors [35,36], a result of weight loss. At baseline, all participants in our study had HOMA-IR indexes >4, which were improved at the end of the study. Moreover, changes in HOMA-IR were associated with improved VAI in both diets and FLI, lipid accumulation products, and triglycerides in the Med diet group, as shown by the regression analysis. Thus, the HOMA-IR index is a useful method for not only diagnosing insulin resistance but also to follow-up the effects of treatments on patients with NAFLD. The evidence from recent clinical and epidemiological studies strongly support the concept that NAFLD is the hepatic manifestation of the metabolic syndrome [37,38].

Due to lower amounts of SFAs, the Med diet, unlike the low-fat diet, induced a significant decrease of 16:0 and total SFAs in serum phospholipids. SFA accumulation in plasma and liver is a general hallmark of NAFLD, causing direct and indirect toxic effects, including altered lipid homeostasis, disruption of desaturase activities, and hepatocyte injury [39,40]. In addition, the Med diet is rich in *n*-3 PUFAs, which promote essential FA desaturation and elongation, leading to increased utilization and lower levels of proinflammatory arachidonic acid [41]. Both diets increased EPA, DHA, and total *n*-3 PUFA levels and decreased the *n*-6/*n*-3 ratio, resulting in an improvement of serum phospholipid FA profiles. As *n*-3 PUFAs are precursors for anti-inflammatory eicosanoids, their dietary-induced uprise diminishes hepatotoxic alternations in NAFLD [40]. The reduced *n*-6/*n*-3 ratio found in both study groups could be related to reduced hepatic steatosis. In addition, an elevated *n*-6/*n*-3 ratio is associated with attenuated lipid oxidation and secretion and increased lipid accumulation in the liver [41,42]. Even more, PUFAs can be enzymatically released from membrane phospholipids [41]. Then, free long-chain *n*-3 PUFAs act as inhibitors of lipogenic and glycolytic genes and/or activators of genes involved in lipid oxidation, contributing to the beneficial effects of the diets. NAFLD is also characterized by impaired activity of key enzymes in PUFA biosynthesis [16]. Thus, the higher increase of DHA levels in NAFLD patients after the Med diet compared to the low-fat diet group may indicate a greater efficacy of the Med diet to restore Δ-5 and Δ-6 FA desaturase activities. Due to increased olive oil consumption, the Med diet also provides more MUFAs than the low-fat diet, which results in higher proportions of oleic acid and total MUFAs in serum phospholipids. Through directly affecting the synthesis of various antioxidative enzymes, increased intakes of oleic acid may reduce liver tissue damage generated by oxidative stress [43]. Thus, protection from MUFAs depletion is another way for NAFLD patients to benefit from the Med diet; favorable changes in FA profiles are significantly higher after the Med diet than after the low-fat diet.

Since approximately 5 months are needed for erythrocyte FA composition to reach saturation [44], a 3-month dietary treatment was insufficient to induce similar changes in erythrocytes as found in serum phospholipids. Nevertheless, some changes and trends can be seen even after the 3-month intervention [45,46]. Here, we found increased oleic acid, MUFAs, EPA, DHA, and *n*-3 PUFAs, as well as MUFA/SFA and decreased *n*-6/*n*-3 PUFAs ratio in the Med diet group after the intervention period. In the low-fat group, there were higher levels of DHA, *n*-3 PUFAs, and a reduced *n*-6/*n*-3 ratio. According to literature data, a dietary-induced erythrocyte DHA enrichment is associated with a reduced liver fat percentage in NAFLD [41]. Additionally, dietary *n*-3 PUFAs modulate lipid metabolism, enhance FA oxidation, and decrease de novo lipogenesis [47]. Therefore, the increased erythrocyte *n*−3 PUFAs found in our study may contribute to NAFLD therapy. In line with this, a recent meta-analysis provided substantial evidence that *n*-3 PUFA supplementation, especially DHA, has a favorable effect in the treatment of NAFLD [17].

This study has some limitations that should be acknowledged. A very large number of NAFLD-related biomarkers have been tested and, therefore, many of the findings may not be independent of each other. Since no penalization of *p*-values has been performed due to multiple comparisons, and the sample size is calculated for a *p* threshold of 0.05, these results should be considered exploratory and further confirmed in future studies. Therefore, the major limitation is the relatively small sample size and relatively short duration of the intervention, especially to detect changes in erythrocyte fatty acid profiles; in spite of this, it can provide a rationale for a large study that would involve more participants and a longer duration. Further, NAFLD was diagnosed using noninvasive ultrasound techniques instead of a liver biopsy, which is the most reliable technique for assessing NAFLD in patients. However, liver biopsy is an invasive method and, thus, unacceptable for many patients, and it is also related to sampling errors and possible procedure-related complications [48]. To mitigate the limitations of the ultrasonography approach, all evaluations were carried out by the same experienced specialist.

The major strengths of this study are that it was a randomized controlled trial and that the adherence to the diets was very high, as regularly checked by phone. Moreover, the personalized approach in dietary counseling and regular, individual follow-ups provided a good basis for long-term lifestyle modifications.

## 5. Conclusions

In summary, our study has shown that lifestyle modifications, mostly dietary calorie-restricted treatments with moderate physical activity, can markedly improve hepatic steatosis, fatty acid profiles, and obesity-related cardiometabolic alterations in NAFLD patients. In particular, the Mediterranean diet was associated with improvements in lipid profiles and fatty liver biochemical indexes, as well as lower saturated fatty acid status and higher levels of monounsaturated and *n*-3 fatty acids. Our results point out the potential utility of this dietary pattern for NAFLD patients. However, future studies should confirm whether these changes are responsible for the benefits of the Med Diet in clinically relevant outcomes in patients with NAFLD.

## Figures and Tables

**Table 1:** Anthropometric and clinical data on nonalcoholic fatty liver disease (NAFLD) patients and the changes induced by dietary treatments.

	Med Diet Baseline (12)	Med Diet End (12)	*p*-Value	Low-Fat Diet Baseline (12)	Low-Fat Diet End (12)	*p*-Value	Low-Fat Diet End vs. Med Diet End *p*-Value
Age (years)	34.42 ± 4.66			32.92 ± 3.78			
Body weight (kg)	101.11 ± 9.09	91.88 ± 9.48	0.000	102.12 ± 8.19	92.41 ± 8.14	0.000	0.342
BMI (kg/m^2^)	30.43 ± 1.81	27.65 ± 1.80	0.000	30.17 ± 2.28	27.68 ± 2.44	0.000	0.342
Waist circumference (cm)	105.67 ± 5.94	95.83 ± 5.73	0.000	107.58 ± 6.96	98.83 ± 8.04	0.000	0.233
Body fat %	26.17 ± 1.71	21.27 ± 3.05	0.000	26.93 ± 3.50	21.86 ± 3.95	0.000	0.989
VAI	2.26 ± 0.81	1.05 ± 0.36	0.000	2.64 ± 0.88	1.52 ± 0.47	0.000	0.083
FLI	81.92 ± 9.95	43.17 ± 7.99	0.000	83.52 ± 10.76	55.08 ± 18.22	0.000	0.022
HSI	47.6 ± 4.92	39.34 ± 3.24	0.000	45.85 ± 4.63	37.67 ± 4.07	0.000	0.435
ultrasonography diagnosed NAFLD	12	6		12	8		

Groups: Med diet baseline and low-fat diet baseline are the initial data; Med diet end and low-fat diet end are the data obtained after 3 months of the respective treatments. Statistics: The values are expressed as means ± SD. Two-tailed Student’s *t*-test for paired data (normally distributed) are used for intragroup comparisons (post- vs. preintervention); For intergroup analyses (low-fat diet end vs. Med diet end), ANCOVA was applied. BW—body weight, BMI—body mass index, VAI—visceral adiposity index, FLI—fatty liver index, HIS—hepatic steatosis index.

**Table 2:** The baseline biochemical serum parameters in NAFLD patients and the changes induced by dietary treatments.

	Med Diet Baseline	Med Diet End	*p*-Value	Low-Fat Diet Baseline	Low-Fat Diet End	*p*-Value	Low-Fat Diet End vs. Med Diet End *p*-Value
Triglycerides (mmol/L)	1.92 (1.35–2.55)	1.06 (0.80–1.20)	0.000 *	2.40 (1.55–2.69)	1.29 (1.20–1.57)	0.000 *	0.048
Total cholesterol (mmol/L)	6.00 ± 0.78	4.83 ± 0.95	0.000	6.08 ± 0.69	4.81 ± 0.82	0.000	0.760
LDL-cholesterol (mmol/L)	3.67 ± 0.72	2.88 ± 0.82	0.000	3.96 ± 0.89	3.14 ± 0.85	0.009	0.857
HDL-cholesterol (mmol/L)	1.29 ± 0.13	1.41 ± 0.15	0.000	1.22 ± 0.12	1.28 ± 0.11	0.041	0.041
TG/HDL-cholesterol ratio,	1.63 ± 0.60	0.78 ± 0.26	0.000	1.87 ± 0.61	1.12 ± 0.36	0.000	0.025
ALT(IU/L)	65.33 ± 23.90	27.33 ± 6.46	0.000	63.17 ± 15.76	31.92 ± 11.89	0.000	0.128
AST(IU/L)	32.50 (23.00–32.75)	20.00 (16.00–21.75)	0.000 *	35.65 (25.00–41.50)	25.50 (18.75–30.75)	0.006 *	0.017
GGT(IU/L)	47.42 ± 36.25	24.33 ± 11.57	0.000	42.53 ± 10.48	27.08 ± 9.90	0.000	0.224
Fasting glucose (mmol/L)	5.11 ± 0.51	4.49 ± 0.68	0.000	5.20 ± 0.45	4.84 ± 0.48	0.032	0.145
Fasting insulin (pmol/L)	17.70 ± 3.24	13.67 ± 2.94	0.000	17.33 ± 3.76	14.38 ± 3.17	0.006	0.475
HOMA-IR	3.96 (3.40−4.76)	2.63 (2.28–3.04)	0.000 *	4.07 (3.45–4.43)	2.86 (2.53–3.37)	0.000 *	0.142
hs-CRP (mg/L)	1.02 (0.75–2.23)	0.81 (0.34–1.40)	0.000 *	2.10 (0.98–3.20)	0.77 (0.54–1.27)	0.008 *	0.239
LAP	78.44 ± 27.40	34.66 ± 16.78	0.000	94.58 ± 33.92	46.63 ± 12.51	0.000	0.032

Groups: Med diet baseline and low-fat diet baseline are the initial data; Med diet end and low-fat diet end are the data obtained after 3 months of the respective treatments. Statistics: The values are expressed as means ± SD for normally distributed variables and median values (25th–75th percentiles) for non-normally distributed variables. Two-tailed Student’s *t*-test for paired data (normally distributed) and the Wilcoxon test (nonparametric data, assigned with *) are used for intragroup comparisons (post- vs. preintervention). For intergroup analyses (low-fat diet end vs. Med diet end, * *p* ≤ 0.05), ANCOVA was applied. hs-CRP—C reactive protein, LAP—lipid accumulation product, TG- Triglycerides

**Table 3:** Serum phospholipid fatty acid composition in NAFLD patients at baseline and after the dietary treatments.

Fatty Acid (%)	Med Diet Baseline	Med Diet End	*p*-Value	Low-Fat Diet Baseline	Low-Fat Diet End	*p*-Value	Low-Fat Diet End vs. Med Diet End *p*-Value
16:0	30.18 ± 3.33	27.58 ± 1.89	0.017	30.32 ± 2.93	29.94 ± 2.59	0.404	0.038
18:0	16.09 ± 2.01	15.47 ± 1.32	0.561	16.40 ± 1.21	15.92 ± 2.26	0.408	0.236
SFA	46.26 ± 4.04	43.05 ± 1.73	0.036	46.72 ± 2.24	45.86 ± 2.73	0.237	0.020
16:1 *n*-7	0.36 ± 0.11	0.41 ± 0.15	0.210	0.42 ± 0.17	0.36 ± 0.07	0.329	0.208
18:1 *n*-9	8.37 ± 1.60	10.58 ± 2.56	0.049	9.80 ± 1.33	8.57 ± 0.78	0.000	0.048
18:1 *n*-7	1.56 ± 0.32	1.57 ± 0.65	0.942	1.17 ± 0.57	1.43 ± 0.44	0.403	0.597
MUFA	10.28 ± 1.66	12.57 ± 2.18	0.033	11.39 ± 1.42	10.36 ± 0.95	0.037	0.011
18:2	23.91 ± 3.69	21.49 ± 3.97	0.040	22.44 ± 3.32	22.69 ± 2.89	0.055	0.125
20:3	3.08 ± 0.76	2.63 ± 1.00	0.341	3.45 ± 0.68	2.87 ± 0.94	0.605	0.809
20:4	12.15 ± 1.35	12.10 ± 1.81	0.936	11.49 ± 1.90	10.85 ± 1.57	0.389	0.237
22:4	0.63 (0.51−0.94)	0.62 (0.41–1.11)	0.865 *	0.60 (0.36–0.81)	0.44 (0.25–0.80)	0.605 *	0.455
*n*-6 PUFA	40.02 ± 4.36	37.06 ± 4.26	0.111	37.96 ± 2.86	36.93 ± 3.53	0.119	0.587
18:3	0.22 (0.07–0.33)	0.12 (0.09–0.32)	0.879 *	0.17 (0.13–0.25)	0.26 (0.13–0.40)	0.176 *	0.696
20:5	0.22 (0.20–0.26)	0.39 (0.25–0.53)	0.030 *	0.38 (0.28–0.71)	0.88 (0.44–1.56)	0.047 *	0.425
22:5	0.61 ± 0.23	0.69 ± 0.27	0.288	0.66 ± 0.12	0.76 ± 0.16	0.101	0.757
22:6	2.67 ± 0.95	5.17 ± 1.19	0.002	2.56 ± 0.77	3.96 ± 1.02	0.004	0.050
*n*-3 PUFA	3.74 ± 1.27	6.53 ± 1.40	0.002	3.87 ± 1.04	5.92 ± 1.71	0.005	0.397
PUFA	43.76 ± 4.22	43.59 ± 3.97	0.987	41.83 ± 2.42	42.85 ± 3.67	0.189	0.977
*n*-6/*n*-3	11.92 ± 4.35	5.94 ± 1.49	0.003	10.53 ± 3.44	6.74 ± 2.14	0.004	0.245
MUFA/SFA	0.22 ± 0.41	0.29 ± 1.26	0.000	0.24 ± 0.03	0.23 ± 0.03	0.138	0.004
MUFA/*n*-6	0.26 ± 0.38	0.34 ± 0.51	0.009	0.30 ± 0.05	0.28 ± 0.01	0.172	0.079
MUFA/*n*-3	2.75 ± 1.30	1.92 ± 1.56	0.023	3.13 ± 0.90	1.90 ± 0.63	0.000	0.576
MUFA/PUFA	0.22 (0.20–0.26)	0.25 (0.25–0.33)	0.099 *	0.26 (0.24–0.31)	0.23 (0.21–0.26)	0.015 *	0.089

Groups: Med diet baseline and low-fat diet baseline are the initial data; Med diet end and low-fat diet end are the data obtained after 3 months of the respective treatments. Statistics: The values are expressed as means ± SD for normally distributed variables and median values (25th–75th percentiles) for non-normally distributed variables. Two-tailed Student’s *t*-test for paired data (normally distributed) and the Wilcoxon test (nonparametric data, assigned with *) are used for intragroup comparisons (post- vs. preintervention). For intergroup analyses (low-fat diet end vs. Med diet end), ANCOVA was applied. SFA—saturated fatty acid, MUFA—monounsaturated fatty acid, PUFA—polyunsaturated fatty acid.

**Table 4:** Erythrocyte phospholipid fatty acid composition in NAFLD patients at baseline and after the dietary treatments.

Fatty Acid (%)	Med Diet Baseline	Med Diet End	*p*-Values	LF Diet Baseline	LF Diet End	*p*-Values	Low-Fat Diet End vs. Med Diet End *p*-Value
16:0	23.24 ± 2.33	22.74 ± 2.23	0.436	25.29 ± 1.74	24.62 ± 1.06	0.344	0.296
18:0	20.50 ± 1.48	20.08 ± 1.29	0.627	21.91 ± 1.35	21.31 ± 2.78	0.521	0.353
SFA	43.74 ± 3.21	42.82 ± 2.11	0.447	47.20 ± 2.40	45.93 ± 3.29	0.243	0.225
16:1 *n*-7	0.26 ± 0.13	0.28 ± 0.11	0.575	0.30 ± 0.15	0.31 ± 0.10	0.845	0.694
18:1 *n*-9	14.03 ± 1.39	15.53 ± 1.20	0.002	14.45 ± 1.85	15.10 ± 2.08	0.256	0.222
18:1 *n*-7	1.14 ± 0.24	1.36 ± 0.28	0.138	1.49 ± 0.52	1.49 ± 0.25	0.911	0.724
MUFA	15.44 ± 1.48	17.17 ± 1.37	0.002	16.25 ± 1.90	16.89 ± 2.24	0.208	0.144
18:2	14.02 ± 1.38	12.92 ± 1.49	0.158	12.67 ± 2.08	11.91 ± 2.42	0.209	0.795
20:3	1.71 ± 0.38	1.55 ± 0.31	0.328	1.52 ± 0.32	1.36 ± 0.30	0.232	0.341
20:4	16.00 ± 2.02	16.20 ± 2.45	0.858	14.19 ± 1.17	14.40 ± 1.25	0.677	0.179
22:4	4.02 (3.32–4.64)	3.62 (3.29–4.37)	0.956 *	3.11 (2.42–4.94)	3.27 (2.50–4.95)	0.564 *	0.671
*n*-6 PUFA	35.78 ± 1.87	34.73 ± 3.15	0.477	31.90 ± 2.37	31.39 ± 2.66	0.644	0.113
18:3	0.07 (0.05–0.28)	0.11 (0.05–0.33)	0.438 *	0.07 (0.04–0.35)	0.09 (0.06–0.27)	0.555 *	0.935
20:5	0.29 (0.27–0.39)	0.45 (0.29–0.83)	0.112 *	0.29 (0.15–0.45)	0.32 (0.23–0.53)	0.992 *	0.183
22:5	1.43 ± 0.43	1.68 ± 0.50	0.029	1.07 ± 0.41	1.29 ± 0.32	0.154	0.426
22:6	3.17 ± 0.75	4.12 ± 1.14	0.028	2.81 ± 0.95	3.69 ± 1.34	0.015	0.855
*n*-3 PUFA	5.04 ± 1.24	6.50 ± 1.72	0.011	4.40 ± 1.55	5.53 ± 1.82	0.021	0.554
PUFA	40.82 ± 2.54	41.24 ± 3.92	0.800	36.30 ± 3.22	36.92 ± 3.16	0.489	0.237
*n*-6/*n*-3	7.41 (6.72–8.64)	5.98 (4.41–6.50)	0.004 *	7.31 (6.31–9.58)	5.04 (4.32–8.82)	0.041 *	0.679
MUFA/SFA	0.35 ± 0.46	0.40 ± 0.03	0.005	0.34 ± 0.04	0.37 ± 0.06	0.132	0.215
MUFA/*n*-6	0.43 ± 0.79	0.50 ± 0.07	0.029	0.51 ± 0.09	0.54 ± 0.08	0.272	0.824
MUFA/*n*-3	3.16 (2.83–3.62)	2.67 (2.24–3.44)	0.165 *	3.62 (2.76–4.95)	2.92 (2.26–5.38)	0.079 *	0.914
MUFA/PUFA	0.38 ± 0.58	0.42 ± 0.06	0.095	0.45 ± 0.09	0.46 ± 0.08	0.617	0.709

Groups: Med diet baseline and low-fat diet baseline are the initial data; Med diet end and Low-fat diet end are the data obtained after 3 months of the respective treatments. Statistics: The values are expressed as means ± SD for normally distributed variables and median values (25th–75th percentiles) for non-normally distributed variables. Two-tailed Student’s *t*-test for paired data (normally distributed) and the Wilcoxon test (nonparametric data, assigned with *) are used for intragroup comparisons (post- vs. preintervention). For intergroup analyses (low-fat diet end vs. Med diet end), ANCOVA was applied. SFA—saturated fatty acid, MUFA—monounsaturated fatty acid, PUFA—polyunsaturated fatty acid.

**Table 5:** Multiple regression analysis of the relationship of **ΔFLI** with the type of diet (low-fat or Med diet) adjusted for age, AST, HDL-cholesterol, and triglycerides.

	ΔFLI	β	*p*	Adjusted R^2^
Model 1	Low-fat diet vs. Med diet	0.377	0.043	0.103
Model 2	Low-fat diet vs. Med diet	0.452	0.023	0.243
	Age	−0.115	0.235	
Model 3	Low-fat diet vs. Med diet	0.466	0.032	0.273
	ΔAST	0.010	0.964	
	ΔHDL	−0.373	0.131	
	ΔTG	−0.256	0.251	

Model 2 was adjusted by age, and Model 3 was adjusted by AST, HDL-cholesterol, and TG. FLI—fatty liver index; TG—triglycerides; Δ—the differences from study end to baseline. R^2^ represents the coefficient of determination and β the standardized regression coefficient.

## Data Availability

The data presented in this study are available on request from the corresponding author. The data are not publicly available due to or ethical reason.

## Supplementary Materials

The following are available online at https://www.mdpi.com/2072-6643/13/1/15/s1, Table S1: Summary of Dietary Recommendations to Participants and Compositions of Mediterranean diet and Low-fat diet, Table S2: Univariate linear regression analysis of the markers of hepatic status after 3-months dietary intervention with Med diet of Low-fat diet (dependent varibles) and changes in anthropometric and biochemical parameters (independent variables).

## References

[1] Portillo-Sanchez P., Cusi K. (2016). Treatment of nonalcoholic fatty liver disease (NAFLD) in patients with type 2 diabetes mellitus. Clin. Diabetes Endocrinol..

[2] Chalasani N., Younossi Z., Lavine J.E., Charlton M., Cusi K., Rinella M., Harrison S.A., Brunt E.M., Sanyal A.J. (2018). The diagnosis and management of nonalcoholic fatty liver disease: Practice guidance from the American association for the study of liver diseases. Hepatology.

[3] Pan J.J., Fallon M.B. (2014). Gender and racial differences in nonalcoholic fatty liver disease. World J. Hepatol..

[4] Kawano Y., Cohen D.E. (2013). Mechanisms of hepatic triglyceride accumulation in non-alcoholic fatty liver disease. J. Gastroenterol..

[5] Sporea I., Popescu A., Dumitrașcu D., Brisc C., Nedelcu L., Trifan A., Gheorghe L., Fierbințeanu Braticevici C. (2018). Nonalcoholic fatty liver disease: Status quo. J. Gastrointestin. Liver. Dis..

[6] Trovato F.M., Catalano D., Martines G.F., Pace P., Trovato G.M. (2015). Mediterranean diet and non-alcoholic fatty liver disease: The need of extended and comprehensive interventions. Clin. Nutr..

[7] Promrat K., Kleiner D.E., Niemeier H.M., Jackvony E., Kearns M., Wands J.R., Fava J.L., Wing R.R. (2010). Randomized controlled trial testing the effects of weight loss on nonalcoholic steatohepatitis. Hepatology.

[8] Sorrentino G., Crispino P., Coppola D., De Stefano G. (2015). Efficacy of lifestyle changes in subjects with non-alcoholic liver steatosis and metabolic syndrome may be improved with an antioxidant nutraceutical: A controlled clinical study. Drugs RD.

[9] Marchesini G., Petta S., Dalle Grave R. (2016). Diet, weight loss, and liver health in nonalcoholic fatty liver disease: Pathophysiology, evidence, and practice. Hepatology.

[10] Plaz Torres M., Aghemo A., Lleo A., Bodini G., Furnari M., Marabotto E., Miele L., Giannini E. (2019). Mediterranean diet and NAFLD: What we know and questions that still need to be answered. Nutrients.

[11] Abenavoli L., Milic N., Peta V., Alfieri F., De Lorenzo A., Bellentani S. (2014). Alimentary regimen in non-alcoholic fatty liver disease: Mediterranean diet. World J. Gastroenterol..

[12] Ferramosca A., Zara V. (2014). Modulation of hepatic steatosis by dietary fatty acids. World J. Gastroenterol..

[13] Tetri L.H., Basaranoglu M., Brunt E.M., Yerian L.M., Neuschwander-Tetri B.A. (2008). Severe NAFLD with hepatic necroinflammatory changes in mice fed trans fats and a high fructose corn syrup equivalent. Am. J. Physiol. Gastrointest. Liver Physiol..

[14] Di Minno M.N., Russolillo A., Lupoli R., Ambrosino P., Di Minno A., Tarantino G. (2012). Omega-3 fatty acids for the treatment of non-alcoholic fatty liver disease. World J. Gastroenterol..

[15] López-Bautista F., Barbero-Becerra V.J., Ríos M.Y., Ramírez-Cisneros M.Á., Sánchez-Pérez C.A., Ramos-Ostos M.H., Uribe M., Chávez-Tapia N.C., Juárez-Hernández E. (2020). Dietary consumption and serum pattern of bioactive fatty acids in NAFLD patients. Annals Hepatol..

[16] Elizondo A., Araya J., Rodrigo R., Signorini C., Sgherri C., Comporti M., Poniachik J., Videla L.A. (2008). Effects of weight loss on liver and erythrocyte polyunsaturated fatty acid pattern and oxidative stress status in obese patients with non-alcoholic fatty liver disease. Biol. Res..

[17] Guo X., Yang B., Tang J., Li D. (2018). Fatty acid and non-alcoholic fatty liver disease: Meta-analyses of case-control and randomized controlled trials. Clin. Nutr..

[18] Vernon G., Baranova A., Younossi Z.M. (2011). Systematic review: The epidemiology and natural history of non-alcoholic fatty liver disease and nonalcoholic steatohepatitis in adults. Aliment. Pharmacol. Ther..

[19] Trumbo P., Schlicker S., Yates A.A., Poos M. (2002). Dietary reference intakes for energy, carbohydrate, fiber, fat, fatty acids, cholesterol, protein and amino acids. J. Am. Diet. Assoc..

[20] Amato M.C., Giordano C., Galia M., Criscimanna A., Vitabile S., Midiri M., Galluzzo A. (2010). Visceral adiposity index: A reliable indicator of visceral fat function associated with cardiometabolic risk. Diabetes Care.

[21] Keskinler V.M., Mutlu H., Sirin A., Senates E.B., Colak Y., Tuncer I., Oguz A. (2020). Visceral adiposity index as a practical tool in patients with biopsy-proven nonalcoholic fatty liver disease/nonalcoholic steatohepatitis. Metab. Syndr. Relat. Disord..

[22] Gastaldelli A., Kozakova M., Hojlund K., Flyvbjerg A., Favuzzi A., Mitrakou A., Balkau B. (2009). The RISC investigators. Fatty liver is associated with insulin resistance, risk of coronary heart disease, and early atherosclerosis in a large European population. Hepatology.

[23] Lee J.H., Kim D., Kim H.J., Lee C.H., Yang J.I., Kim W., Kim Y.J., Yoon J.-H., Cho S.-H., Sung M.-W. (2010). Hepatic steatosis index: A simple screening tool reflecting nonalcoholic fatty liver disease. Dig. Liver. Dis..

[24] Bedogni G., Kahn H.S., Bellentani S., Tiribelli C. (2010). A simple index of lipid over accumulation is a good marker of liver steatosis. BMC Gastroenterol..

[25] Ristić-Medić D., Ristić V., Tepšić V., Ranić M., Ristić G., Vrbaški S., Estelecki I. (2003). Effect of soybean Leci-Vita product on serum lipids and fatty acid composition in patients with elevated serum cholesterol and triglyceride levels. Nutr. Res..

[26] Musso G., Cassader M., Rosina F., Gambino R. (2012). Impact of current treatments on liver disease, glucose metabolism and cardiovascular risk in nonalcoholic fatty liver disease (NAFLD): Systematic review and meta-analysis of randomised trials. Diabetologia.

[27] Abenavoli L., Greco M., Milic N., Accattato F., Foti D., Gulletta E., Luzza F. (2017). Effect of Mediterranean diet and antioxidant formulation in non-alcoholic fatty liver disease: A randomized study. Nutrients.

[28] Goldberg I.J., Ginsberg H.N. (2006). Ins and outs modulating hepatic triglyceride and development of nonalcoholic fatty liver disease. Gastroenterology.

[29] Fabbrini E., Mohammed B.S., Magkos F., Korenblat K.M., Patterson B.W., Klein S. (2008). Alterations in adipose tissue and hepatic lipid kinetics in obese men and women with nonalcoholic fatty liver disease. Gastroenterology.

[30] Min H.K., Kapoor A., Fuchs M., Mirshahi F., Zhou H., Maher J., Kellum J., Warnick R., Contos M.J., Sanyal A.J. (2012). Increased hepatic synthesis and dysregulation of cholesterol metabolism is associated with the severity of nonalcoholic fatty liver disease. Cell Metab..

[31] Trojak A., Waluś-Miarka M., Woźniakiewicz E., Małecki M.T., Idzior-Waluś B. (2013). Nonalcoholic fatty liver disease is associated with low HDL cholesterol and coronary angioplasty in patients with type 2 diabetes. Med. Sci. Monit..

[32] DeFilippis A.P., Blaha M.J., Martin S.S., Reed R.M., Jones S.R., Nasir K., Blumenthal R.S., Budoff M.J. (2013). Nonalcoholic fatty liver disease and serum lipoproteins: The multi-ethnic study of atherosclerosis. Atherosclerosis.

[33] Sofi F., Casini A. (2014). Mediterranean diet and non-alcoholic fatty liver disease: New therapeutic option around the corner?. World J. Gastroenterol..

[34] Finelli C., Tarantino G. (2012). Is there any consensus as to what diet or lifestyle approach is the right one for NAFLD patients?. J. Gastrointestin. Liver Dis..

[35] Velasco N., Contreras A., Grassi B. (2014). The mediterranean diet, hepatic steatosis and nonalcoholic fatty liver disease. Curr. Opin. Clin. Nutr. Metab. Care..

[36] Ryan M.C., Itsiopoulos C., Thodis T., Ward G., Trost N., Hofferberth S., O’Dea K., Desmond P.V., Johnson N.A., Wilson A.M. (2013). The Mediterranean diet improves hepatic steatosis and insulin sensitivity in individuals with non-alcoholic fatty liver disease. J. Hepatol..

[37] Abenavoli L., Milic N., Di Renzo L., Preveden T., Medić-Stojanoska M., De Lorenzo A. (2016). Metabolic aspects of adult patients with nonalcoholic fatty liver disease. World J. Gastroenterol..

[38] Gentile C.L., Pagliassotti M.J. (2008). The role of fatty acids in the development and progression of nonalcoholic fatty liver disease. J. Nut. Biochem..

[39] Wang X., Cao Y., Fu Y., Guo G., Zhang H. (2011). Liver fatty acid composition in mice with or without nonalcoholic fatty liver disease. Lipids Health Dis..

[40] Araya J., Rodrigo R., Videla L.A., Thielemann L., Orellana M., Pettinelli P., Poniachik J. (2004). Increase in long-chain polyunsaturated fatty acid n-6/n-3 ratio in relation to hepatic steatosis in patients with non-alcoholic fatty liver disease. Clin. Sci..

[41] Juarez-Hernandez E., Chavez-Tapial N.C., Uribe M., Barbero-Becerra V.J. (2016). Role of bioactive fatty acids in nonalcoholic fatty liver disease. Nutr. J..

[42] Hwang J., Chang Y.H., Park J.H., Kim S.Y., Chung H., Shim E., Hwang H.J. (2011). Dietary saturated and monounsaturated fats protect against acute acetaminophen hepatotoxicity by altering fatty acid composition of liver microsomal membrane in rats. Lipids Health Dis..

[43] Bhattacharjee B., Pal P., Chattopadhyay A., Bandyopadhyay D. (2020). Oleic acid protects against cadmium induced cardiac and hepatic tissue injury in male Wistar rats: A mechanistic study. Life Sci..

[44] De Castro G.S., Calder P.C. (2018). Non-alcoholic fatty liver disease and its treatment with n-3 polyunsaturated fatty acids. Clin. Nutr..

[45] Guebre-Egziabher F., Rabasa-Lhoret R., Bonnet F., Bastard J.P., Desage M., Skilton M.R., Vidal H., Laville M. (2008). Nutritional intervention to reduce the n-6/n-3 fatty acid ratio increases adiponectin concentration and fatty acid oxidation in healthy subjects. Eur. J. Clin. Nutr..

[46] Seethaler B., Basrai M., Vetter W., Lehnert K., Engel C., Siniatchkin M., Halle M., Kiechle M., Bischoff S.C. (2020). Fatty acid profiles in erythrocyte membranes following the Mediterranean diet-data from a multicenter lifestyle intervention study in women with hereditary breast cancer (LIBRE). Clin. Nutr..

[47] Green C.J., Pramfalk C., Charlton C.A., Gunn P.J., Cornfield T., Pavlides M., Karpe F., Hodson L. (2020). Hepatic de novo lipogenesis is suppressed and fat oxidation is increased by omega-3 fatty acids at the expense of glucose metabolism. BMJ Open Diabetes Res. Care.

[48] Marin-Alejandre B.A., Abete I., Cantero I., Monreal J.I., Elorz M., Herrero J.I., Huarte-Muniesa M.P. (2019). The metabolic and hepatic impact of two personalized dietary strategies in subjects with obesity and nonalcoholic fatty liver disease: The fatty liver in obesity (FLiO) randomized controlled trial. Nutrients.

