# Supplementary material for: Calorie-Restricted Mediterranean and Low-Fat Diets Affect Fatty Acid Status in Individuals with Nonalcoholic Fatty Liver Disease
Source: Nutrients. 2020 Dec 23;13(1):15. doi: 10.3390/nu13010015 (PMC7822481; doi:10.3390/nu13010015)
Supplement: Supplementary file 1 [file nutrients-13-00015-s001.pdf]

Table S1. Summary of Dietary Recommendations to Participants and Compositions of Mediterranean diet and low-fat diet.

Mediterranean diet		Low-fat diet	
carbohydrates	50% en	carbohydrates	60% en
proteins	15% en	proteins	15% en
total fats	>30% en	total fats	≤25% en
saturated	<8% en	saturated	<10% en
monounsaturat	>15% en	monounsaturated	>10% en
polyunsaturated	>5% en	polyunsaturated	>5 % en
**Recommended**	**Goal**	**Recommended**	**Goal**
Fresh fruit	3 serving/day	Fresh fruit	3 servings/day
Vegetable	2 servings/day	Vegetable	2 servings/day
Nuts	20 g/daily (10g walnuts, 5 g hazelnuts, 5 g almonds)	Low fat dairy products	3 servings/day
		Bread, potatoes, pasta, rice	3 servings/day
Whole grain (legumes, rice, maize, wheat)	300 g/day	Lean fish and seafood	2 servings/week
Olive oil	Main source of fat ≥4 tablespoons/day	**Discouraged**	**Goal**
Fish (especially fatty fish), seafood	≥300g/ week	Vegetable oils (including olive oil)	≤2 tablespoons/day
White meat	Instead of red meat	Spread fats	≤1serving/week
**Discouraged**	**Goal**	Red and processed fatty meat	≤1serving/week
Soda drinks	≤1 drink/day	Fatty fish, seafood canned in oil	1serving/week
Commercial bakery products, sweets and pastries	≤3serving/week	Commercial bakery products, sweets and pastries	≤1serving/week
Spread fats	≤1serving/day		
Red and processed meats	≤1serving/day	Nuts and fried snacks	≤1serving/week
**Not allowed**			
butter			
sour cream			
lard			

Table S2. Univariate linear regression analysis of the markers of hepatic status after 3-months dietary intervention with Med diet of Low-fat diet (dependent varibles) and changes in anthropometric and biochemical parameters (independent variables).

	Linear Regression Analysis															
	Mediterranean diet															
	**Independent variables (predictors)**	**ΔBMI (kg/m²)**			**Δ Body fat %**			**ΔWC (cm)**			**ΔHOMAIR**			**Δ hs-CRP (mg/l)**		
		**B**	***β***	***p***	**B**	***β***	***p***	**B**	***β***	***p***	**B**	***β***	***p***	**B**	***β***	***p***
**Dependent variable**	**VAI**	0.353	0.867	0.150	−0.178	−1.414	0.025	0.038	0.205	0.593	−0.321	−0.877	0.016	0.104	0.100	0.710
	**LAP**	20.608	1.088	0.087	−7.567	−1.294	0.037	−1.511	−0.175	0.651	−14.103	−0.827	0.021	−1.543	−0.032	0.906
	**HSI**	−1.941	−0.530	0.607	0.553	0.489	0.602	0.087	0.052	0.941	0.586	0.178	0.729	−1.059	−0.114	0.819
	**FLI**	11.102	0.547	0.322	−6.395	−1.020	0.048	0.429	0.046	0.899	−16.528	−0.904	0.012	−1.009	−0.020	0.939
	**AST(IU/L)**	3.938	2.990	0.236	−1.809	−1.298	0.044	−0.069	−0.034	0.944	−1.991	−0.490	0.190	−0.848	−0.074	0.826
	**ALT(IU/L)**	2.258	0.310	0.734	−1.098	−0.488	0.560	−1.201	−0.362	0.569	−0.009	−0.001	0.998	−2.575	−0.139	0.754
	**TG (mmol/l)**	0.405	0.936	0.176	−0.149	−1.113	0.041	−0.036	−0.184	0.677	−0.314	−0.807	0.038	−0.005	−0.005	0.988
	**Low-fat diet**															
**Dependent variable**	**VAI**	−0.241	−0.286	0.349	−0.135	−0.428	0.159	0.051	0.205	0.457	−0.769	−0.533	0.049	−0.149	−0.121	0.630
	**LAP**	−3.446	−0.286	0.480	−1.588	−0.352	0.366	−0.294	−0.084	0.818	−1.253	−0.061	0.858	3.036	0.174	0.609
	**HSI**	1.996	0.511	0.113	−0.348	−0.237	0.396	−0.821	−0.717	0.029	1.020	0.153	0.541	1.960	0.346	0.189
	**FLI**	−1.160	−0.066	0.860	−2.003	−0.304	0.405	−2.378	−0.463	0.208	0.726	0.024	0.940	3.638	0.143	0.655
	**AST(IU/L)**	−3.497	−0.402	0.159	3.147	0.965	0.007	−0.363	−0.142	0.557	−0.781	−0.053	0.815	2.671	0.212	0.358
	**ALT(IU/L)**	2.182	0.191	0.551	2.842	0.662	0.039	−1.960	−0.585	0.028	−1.529	−0.078	0.773	6.727	0.406	0.165
	**TG (mmol/l)**	−0.108	−0.290	0.495	−0.044	−0.315	0.436	0.047	0.434	0.280	−0.143	−0.225	0.536	−0.120	−0.223	0.535

BMI- body mass index, WC-waist circumference, hs-CRP – C reactive protein, VAI-visceral adiposity index, LAP – lipid accumulation products, FLI -fatty liver index, HSI-hepatic steatosis index. TG-triglycerides.
